# On manipulation by emotional AI: UK adults’ views and governance implications

**DOI:** 10.3389/fsoc.2024.1339834

**Published:** 2024-06-07

**Authors:** Vian Bakir, Alexander Laffer, Andrew McStay, Diana Miranda, Lachlan Urquhart

**Affiliations:** ^1^School of History, Law and Social Sciences, Bangor University, Bangor, United Kingdom; ^2^School of Media and Film, University of Winchester, Winchester, United Kingdom; ^3^Faculty of Social Sciences, University of Stirling, Scotland, United Kingdom; ^4^Edinburgh Law School, University of Edinburgh, Scotland, United Kingdom

**Keywords:** biometrics, digital manipulation, emotional AI, emotion profiling, emotoys, governance, manipulation, public opinion

## Abstract

With growing commercial, regulatory and scholarly interest in use of Artificial Intelligence (AI) to profile and interact with human emotion (“emotional AI”), attention is turning to its capacity for *manipulating* people, relating to factors impacting on a person’s decisions and behavior. Given prior social disquiet about AI and profiling technologies, surprisingly little is known on people’s views on the benefits and harms of emotional AI technologies, especially their capacity for manipulation. This matters because regulators of AI (such as in the European Union and the UK) wish to stimulate AI innovation, minimize harms and build public trust in these systems, but to do so they should understand the public’s expectations. Addressing this, we ascertain UK adults’ perspectives on the potential of emotional AI technologies for manipulating people through a two-stage study. Stage One (the qualitative phase) uses design fiction principles to generate adequate understanding and informed discussion in 10 focus groups with diverse participants (*n* = 46) on how emotional AI technologies may be used in a range of mundane, everyday settings. The focus groups primarily flagged concerns about manipulation in two settings: emotion profiling in social media (involving deepfakes, false information and conspiracy theories), and emotion profiling in child oriented “emotoys” (where the toy responds to the child’s facial and verbal expressions). In both these settings, participants express concerns that emotion profiling covertly exploits users’ cognitive or affective weaknesses and vulnerabilities; additionally, in the social media setting, participants express concerns that emotion profiling damages people’s capacity for rational thought and action. To explore these insights at a larger scale, Stage Two (the quantitative phase), conducts a UK-wide, demographically representative national survey (*n* = 2,068) on attitudes toward emotional AI. Taking care to avoid leading and dystopian framings of emotional AI, we find that large majorities express concern about the potential for being manipulated through social media and emotoys. In addition to signaling need for civic protections and practical means of ensuring trust in emerging technologies, the research also leads us to provide a policy-friendly subdivision of what is meant by manipulation through emotional AI and related technologies.

## 1 Introduction

Following five decades of research on the sociology of emotions, we have an improved understanding regarding the social nature of emotions and the emotional nature of social reality (Bericat, 2016). The political economy of AI technologies has also developed apace across the past decade, particularly in regard to how emotion is prescribed and defined, including ethical questions about neoliberal interests in defining these answers (Davies, 2015; McStay, 2018, 2023; Stark and Hoey, 2020). Indeed, as well as provoking scholarly interest, the past decade has seen lively commercial and regulatory interest in use of Artificial Intelligence (AI) to profile and interact with human emotion (“emotional AI”). By “emotional AI,” we refer to computers, AI software, sensors and actuators, used to read and react to human emotions and feeling through text, voice, computer vision and biometric sensing, thereby simulating understanding of affect, emotion and intention (McStay, 2018). Advocates of emotional AI point to pro-social usages enabling personalization, better user experience, improved communication, mental gains, personal coaching and educational usages. Conversely, these controversial technologies have attracted critiques on their privacy invasiveness and over-reach into intimate dimensions of human life (McStay, 2016). Biometric forms of emotional AI are also associated with algorithmic racism (Rhue, 2018), physiognomy (Stark and Hutson, 2021), highly questionable accuracy for adults and children (Barrett et al., 2019), and questions regarding the very premise of inferring emotion through human profiling (McStay, 2018, 2023). Attention has also started to turn to emotion profiling technologies’ capacity for manipulating people, as we will discuss in this paper. Given social disquiet about AI and profiling technologies, surprisingly little is known on people’s views on the benefits and harms of emotion profiling technologies, especially their capacity for manipulation. This matters because as regions clamor to decide how to govern and regulate use of AI, yet also allow for national and regional innovation, they should understand the public’s expectations. The goal of this paper is to surface understanding of UK adults’ perspectives on a single aspect of AI (that which uses diverse technical means to profile and interact with emotion) and its potential for manipulating people.

We start by defining manipulation (namely, *denial of autonomy*, or freedom to choose, reflect and deliberate) (see Section 2). Fears have been expressed that manipulation may be surreptitiously achieved in digital environments by deploying profiling and targeting technologies, especially those that access our emotions and inner states. To scrutinize these fears, we examine the two main routes by which emotion profiling has arisen globally: namely, deployment through biometrics, and deployment through psychological inferences made through text, images, and recommender systems programmed to profile emotion. We find that the manipulative impacts of emotion profiling technologies are unknown or unclear, which allows concerns about their manipulative potential to remain. This, alongside growing commercial interest in (and critiques of) emotion profiling technologies, has spurred international and regional regulation in this area.

Most notable is the European Union’s (EU) draft AI Act (first proposed in 2021)—the world’s first comprehensive set of regulations for the AI industry. It takes a risk-based approach to support innovation in AI systems, minimize arising harms, and build trust in AI (European Commission, 2021). The EU’s interest in avoiding manipulation and preserving human autonomy is reflected in the draft AI Act. This is evident both in the version proposed by the Commission [e.g., Recital 16 bans use of “certain AI systems intended to distort human behavior, whereby physical or psychological harms are likely to occur” (European Commission, 2021)], and in various amendments then made to the draft AI Act by the European Parliament in June 2023. For instance, the European Parliament amended Recital 16, so that the prohibition included not just AI systems that intended to distort human behavior, but also those that had that effect regardless of the intention of the provider or the deployer (European Parliament, 2023, amendment 38). The European Parliament also added to the Recitals the text: “artificial intelligence should be a human-centric technology. It should not substitute human autonomy or assume the loss of individual freedom” (European Parliament, 2023, amendment 15). Reflecting this commitment, the European Parliament added emotion recognition systems (by which it means biometrics) to its high-risk list (as well as banning these systems in places such as the workplace and education institutions, although caveats were added for medical and safety reasons). It also added to the high-risk list AI systems to influence voters in political campaigns (amendment 72) (European Parliament, 2023). The implications of being high risk are that the AI manufacturer faces stricter design and development obligations. This includes creating a risk management system, ensuring appropriate data governance is in place, and supporting human oversight of how the system operates. The European Parliament version also adds rules for foundational AI models, particularly image and text-based Generative AI. It requires providers to disclose if outputs are ‘inauthentic’ [European Parliament, 2023, article 28b and article 51(3)(1)]. This seeks to address scope for manipulation and misinformation when it is unclear to consumers if these outputs are genuine, or machine generated.

While the EU’s draft AI Act has been criticized for its vague conceptualization of how manipulation may occur via AI (Franklin et al., 2023), that the Act regards such manipulation as a real threat is clear. In this sense, the draft Act also echoes fears evident in the EU’s landmark Digital Services Act 2022 that stipulates that all online platforms must design, organize, and operate their online interfaces in a way that does not deceive, manipulate, or materially distort or impair users’ ability to make free and informed decisions. It also stipulates that very large online platforms and services must, annually, audit, identify and tackle “systemic risks” arising from the design and use of their services including (a) those that adversely impact fundamental rights or seriously harm users’ physical or mental health; and (b) manipulation of services that impact democratic processes and public security (Council of the EU, 2022). This includes conducting risk assessments, particularly around algorithmic systems used for recommender systems and advertising (Council of the EU, 2022, article 34). Recital 84 of the Digital Services Act also notes concerns that AI can amplify disinformation, misleading and deceptive content, and thus Recital 88 suggests very large online platforms need to mitigate effects of negative recommendations for users.

The UK’s position on AI is more agile, mostly because it does not have law explicitly addressing emotion recognition. However, the UK’s data regulator, the Information Commissioner’s Office, warns against use of high-risk emotional AI technologies on grounds of accuracy, fairness, biases, and scope for discrimination (ICO, 2022). Drawing on critical work by the Emotional AI Lab, the Information Commissioner’s Office also identifies and warns against issues that may fall outside of the scope of data protection (especially applications that are not contingent on uniquely identifying a person) (McStay, 2020a; McStay and Urquhart, 2022a). In June 2023, the UK government updated its White Paper detailing its plan for AI regulation, noting in the Ministerial Foreword to the White Paper that to understand and act on new and emerging AI risks, “A critical component of this activity will be engaging with the public to understand their expectations, raising awareness of the potential of AI and demonstrating that we are responding to concerns” (Department for Science, Innovation, and Technology and Office for Artificial Intelligence, 2023). On the public’s expectations, UK-based nationally representative surveys on attitudes toward a broad range of AI use cases have so far indicated that people have concerns about sale of data for commercial manipulation; and about the potential impact of deepfake technology and profiling technologies on elections and democracy; and that people are more negative about AI where it is seen as replacing (rather than augmenting) human decision-making (Centre for Data Ethics and Innovation, 2022; Ada Lovelace Institute and The Alan Turing Institute, 2023; Luminate, 2023). Yet little is known on whether or why people have concerns about manipulation from emotion profiling.

We address this gap with our two-stage study on the UK public’s views (explaining its methodology in Section 3). This was a broad-based study on UK adults’ views on various facets of emotional AI, including its capacity for good (such as pro-social uses and personalization) and its capacity for harms (such as to privacy, and its potential for bias and manipulation). As emotion profiling is both abstract and complex, ascertaining informed lay views on the issue is difficult. To address this, Stage One of our research (the qualitative phase) uses design fiction techniques to generate informed discussion in focus groups with diverse participants (*n* = 46) on how these technologies may be used in a range of everyday settings (including the home, transport, workplaces, toys, digital platforms, and social media) (see Section 4). Although Stage One was interested in surfacing diverse views (studying participants who are older, younger, identify as disabled or from ethnic minorities), we find that across our diverse focus groups, concerns about manipulation were repeatedly raised in two settings: namely, emotion profiling in social media (involving deepfakes, false information and conspiracy theories), and in child oriented ‘emotoys’ (where the toy responds to the child’s facial and verbal expressions). In both these settings, participants express concerns that emotion profiling covertly exploits users’ cognitive or affective weaknesses and vulnerabilities. Additionally, in the social media setting, participants express concerns that emotion profiling damages people’s capacity for rational thought and action.

In Stage 2, our quantitative stage, we use insights derived from our qualitative Stage 1 to help construct questions for a UK-wide, demographically representative national survey (*n* = 2,068) on attitudes toward emotional AI across 10 different use cases, two of which investigate people’s views on emotional AI’s capacity for manipulation (one such use case involves digital manipulation tools and the other involves emotoys) (see Section 5). Taking care to present positive as well as negative statements for participants to respond to, we find that a majority express concern about the potential for manipulating people.

In Section 6, we discuss our findings, and arising governance implications. We conclude that to build public trust in emotional AI use cases that have potential for manipulating people, there is a need for strong social protections, in line with our paper’s clarification of the two types of manipulation that concern people.

## 2 Undermining autonomy

We show below (Section 2.1) that while scholars from diverse disciplines highlight different components of manipulation, they agree that manipulation comprises *denial of autonomy*. They fear that manipulation may be surreptitiously achieved in digital environments by deploying profiling and targeting technologies, especially those that access our emotions and inner states. To examine this claim, in Section 2.2 we present an overview of the rise of emotion profiling globally, documenting its two main routes. These routes are (a) deployment through biometrics (such as facial coding of expressions, voice analytics or wearables), and (b) deployment through psychological inferences made through text, images, and recommender systems programmed to profile emotion (exemplified by engagement-driven social media platforms). Section 2.3 then discusses studies on users’ views on emotion profiling and manipulation.

### 2.1 Definitions and claims

Scholars from diverse disciplines agree that manipulation involves *undermining of autonomy*, or *freedom to choose, reflect and deliberate* (Sunstein, 2016; Bakir et al., 2019; Susser et al., 2019). In simple terms, this is the ability to freely make up one’s own mind. For instance, coming from communications, sociology and politics perspectives, Bakir et al. (2019) argue that persuasive communications, to avoid being manipulative, should be both *informed* (with *sufficient* information provided, and none of it *deceptive*) and *freely chosen* (namely, no *coercion*, such as threats, and no *incentivization*, such as subsidies). Scholars who study influence and behavior change in the context of ‘nudging’ reach similar conclusions. For instance, legal scholars Susser et al. (2019) define manipulation as using hidden or *covert* means to *subvert another person’s decision-making power*, undermining their autonomy. For them, manipulation involves “exploiting the manipulator’s cognitive (or affective) *weaknesses and vulnerabilities* in order to steer his or her decision-making process toward the manipulator’s ends” (Susser et al., 2019, p. 3). Coming from a policymaking perspective, Sunstein (a proponent of devising policies to “nudge” populations into making better decisions for themselves) defines efforts to influence people’s decision-making choices as manipulative if they do not, “sufficiently engage or appeal to their *capacity for reflection and deliberation*” (Sunstein, 2016, p. 82).

Written at a time when it was assumed that (at least some) humans would be in control of the systems that they create and their manipulative intent, scholars have also pinpointed features of manipulation in the *digital* environment, pointing to opaque *profiling* and *targeting* technologies that could undermine autonomy by modifying human behavior. Although popularized by Zuboff’s (2019) characterization of “surveillance capitalism,” the mechanics of such attempted manipulation were described a decade ago. For instance, based on Skinner’s (2005 [1948]) utopian novel, *Walden Two*, which describes a society built on behavioral engineering, Von Otterlo (2014, p. 257) proposes the metaphor of “Walden 3.0” where communities of interest “can be controlled through various forms of predictive models and manipulations,” that could be exploited to, for example, optimize advertising decisions to influence potential customers. Also published that year, McStay’s (2014) study of behavioral advertising and what he terms the “mood of information” explores contemporary and nascent forms of commercial solicitation predicated on commodification of subjectivity and experience. Beyond commercial manipulation, Susser et al. (2019) regard digital platforms as prime sites of manipulation as widespread digital surveillance allow data collectors and aggregators to identify our weaknesses and leverage those insights on personalized platforms. They argue that digital platforms rob people of autonomy where, for instance, the labor-force is algorithmically nudged, and where psychographic profiling is used to try to influence elections. Bakir (2020), in the context of digital political campaigning, argues that manipulation of the digital media ecology becomes coercive (choice limiting) if it significantly modulates what information people are exposed to in order to preclude reflection or deliberation.

Scholars of human rights have argued that attempts to surreptitiously influence people through such *profiling* and *targeting* technologies may contravene the absolute right to Freedom of Thought, found in article 18 of the 1976 International Covenant on Civil and Political Rights. Alegre, for example, argues that this right protects our mental inner space, as “the concept of ‘thought’ is potentially broad including things such as emotional states, political opinions and trivial thought processes” (Alegre, 2017, p. 224), as well as the right not to have our thoughts manipulated (see also Alegre, 2022). Special mention needs to be made of children too. Although article 5 and article 14 of the United Nations Convention on the Rights of the Child recognize that parents have the right to provide guidance and direction to their child as they grow up, even this does not affect the child’s fundamental civil right to freedom of thought, conscience, and religion (UNCRC, 2013).

Fears abound, then, across multiple scholarly disciplines, that manipulation may be surreptitiously achieved in digital environments by deploying profiling and targeting technologies that have access to our emotions and inner states. Are such fears warranted?

### 2.2 Examining the claims of manipulation through emotion profiling

To examine the claim that manipulation may be surreptitiously achieved by emotion profiling in digital environments, we document below the two main ways in which emotion profiling occurs globally, these being (a) deployment through biometrics and (b) deployment through psychological inferences made through text, images, and recommender systems programmed to profile emotion. For each of these emotion profiling technologies, we examine studies that address manipulation.

Emotion profiling through *biometrics* includes techniques such as facial coding of expressions, voice analytics, eye-tracking, and wearables that sense skin responses, muscle activity, heart activity, respiration, and brain activity. Recent years have seen sector-specific trials of such techniques in security, education and workplaces across the world (McStay, 2018, 2020b, 2023; ARTICLE 19, 2021; Mantello et al., 2021; Urquhart et al., 2022); and biometric (voice-based) emotional AI in voice assistants is also envisaged in the patents of Amazon (the world’s largest online marketplace) to offer users highly tailored services, ads and products from the wider platform (Bakir et al., 2023a). Beyond trials and patents, use of biometrics to gauge emotions is also being rolled out in consumer-facing sectors such as in cars to improve cabin experience and safety (McStay and Urquhart, 2022b); in wearables to help users manage their mental health and day (McStay, 2018); and in robotic toys to adapt and respond to users’ emotions, and to display the toy’s “moods” (McStay and Rosner, 2021). As noted in Section 1, these biometric forms of emotional AI have attracted a multi-faceted critique covering issues such as privacy, racism, accuracy and inferences. Yet, it rarely addresses issues of manipulation. An exception is McStay and Rosner’s (2021) qualitative study of diverse experts on biometric forms of emotional AI in the toy industry in the UK and USA. While this finds scope for positive uses of child nudging, the experts also register concern that technologies that influence emotions are “outright manipulation” of children and their (vulnerable, stressed, worn out or digitally illiterate) parents.

The other key route in which emotional AI has become center stage is through *psychological inferences* made through text, images, and recommender systems programmed to profile emotion. The prime example of this is in engagement-driven social media platforms. For example, Facebook, along with other engagement-driven social media platforms, has long profiled emotion and subjective states, with diverse advertisers and campaigners using their services to try to influence consumers and citizens (Kim et al., 2018; Ong and Cabañes, 2018; Stella et al., 2018; Bakir, 2020; Santini et al., 2021; Papadogiannakis et al., 2022). Studies of social media companies also show that they have long profiled child emotions for profit (Barassi, 2020).

Unlike the literature on emotion profiling via biometrics that rarely studies manipulation, more can be found in the literature on emotion profiling via engagement-driven social media platforms. Some studies explore the platforms’ design features, observing that platforms are structured so that only the most engaging material survives on the platform; with design features that collect and manipulate emotion data about users’ interests to fuel the platforms’ advertising-based business model (Stark, 2018; Vaidhyanathan, 2018; McNamee, 2019). Others examine relationships between algorithmic curation, emotions, and user behavior to explore how they propagate false information online (Corbu et al., 2021; Bakir and McStay, 2022). While a highly complex and opaque area, in 2021 a whistleblower revealed how Facebook’s News Feed machine learning algorithm (that determines what content is prioritized in each user’s Feed) gave outsize weightings to emotional reactions and posts that sparked interactions; that this created communities sharing false, extremist information (Hao, 2021; Oremus et al., 2021); and that this led to skewed (more polarized, more extreme) political offers where political campaigners sought to adapt to the algorithms to reach audiences (Hagey and Horwitz, 2021). The dynamics of such processes is complex and not well understood, not least because of unavailability of platforms’ data about online human behavior, but two recent studies arising from collaboration with Meta (to access data) are instructive. Both studies examine the role of feed algorithms during the 2020 US election, and they find that algorithmic curation has large online effects on engagement and exposure to emotional, polarized, and false content (González-Bailón et al., 2023; Guess et al., 2023a).

Studies are divided on whether such emotionalized online environments influence people’s real-world attitudes on polarization, politics, or belief in false information. Many suggest harmful real-world influence (for instance see Martel et al., 2020; Walter et al., 2020; Van Bavel et al., 2021). For instance, Martel et al.’s (2020) US-based study finds that audience’s reliance on emotion increases their belief in fake news, but this is based on experiments rather than real-world settings. However, more recent studies that link behavioral data on Meta’s platforms with examination of people’s offline political views find minimal impacts. These studies find that changing users’ algorithmic feeds, exposure to reshares, and exposure to content from like-minded sources over a three-month period during the US 2020 election produced large effects on users’ online behavior (as noted above) but had no measurable effects on preregistered attitudinal measures such as affective polarization, ideological extremity, candidate evaluations, and belief in false claims. The authors acknowledge this could be due to the study’s design, including the study’s timing (late in the political campaign) and the impossibility of making inferences from the studies about societal impacts in highly adaptive environments (where demand for certain kinds of content can change the incentives of content producers) (Nyhan et al., 2023; Guess et al., 2023a,b).

Overall, then, the claim that manipulation may be surreptitiously achieved by emotion profiling in digital environments, remains largely unresolved. Moreover, given minimal empirical studies on manipulation via biometrics-based emotion profiling, it is unknown whether its manipulative potential is realizable. However, the many more empirical studies on manipulation via emotion profiling using psychological inferences on social media shows concerted attempts at manipulation for commercial, political, and ideological ends. These exploit, and likely reinforce, the emotional tenor of online environments that studies show are (at least partly) algorithmically emotionalised, although with less clear real-world impacts on people’s attitudes (albeit with some strong recent evidence that impacts may be minimal in political contexts).

### 2.3 Users’ views on emotion profiling and manipulation

Despite several UK-based surveys on attitudes toward a broad range of AI use cases (Centre for Data Ethics and Innovation, 2022; Ada Lovelace Institute and The Alan Turing Institute, 2023; Luminate, 2023), there remains the question of whether, or why, people have concerns about manipulation from emotion profiling. Several more focused studies on people’s views on emotion profiling in social media (Andalibi and Buss, 2020), in emotoys (McStay and Rosner, 2021), and in a wide variety of organizations from healthcare to political campaigners (Bakir et al., 2023b) suggest concerns about the potential for being manipulated via exploitation of their emotion data. However, manipulation has not been the core focus of such studies. Furthermore, studies on users’ views on emotion profiling technologies have been quite restricted in the variety of use cases that they examine and have not included the broad diversity of use cases where emotion profiling (and hence potential for manipulation) can currently be found.

The backdrop painted so far is the rise of emotion profiling across consumer-facing sectors; legal, critical, ethical, and regulatory concerns about potential manipulation; unknown manipulative impacts on users of biometric forms of emotion profiling; and unclear manipulative impacts on users of emotion profiling using psychological inferences on social media, although with clear and large impacts on the digital environment. With little known on whether and why people have concerns about manipulation from emotion profiling, we explain below how we study this in qualitative detail (Stage 1) and quantitative reach (Stage 2).

## 3 Methods

Ascertaining informed lay views on emotion profiling is difficult given the technologies’ abstract, complex, future-facing nature. To that end, stage one of our research (the qualitative phase) used an innovative methodology (combining design fiction principles and the ContraVision technique) to stimulate informed discussion with diverse UK-based participants in focus groups on potential benefits and concerns about emotion profiling. This gave rise to insights that we deployed in the second stage of our research (the quantitative phase) to construct survey questions in a demographically representative national survey (*n* = 2,068 UK adults, online omnibus implemented by survey company ICM Unlimited across 29 June–1 July 2022).

Both stages were conducted online as COVID-19 social distancing restrictions were in place across 2021, and there were health concerns for vulnerable people (such as older adults) in face-to-face situations across 2022. The online format has inclusivity implications regarding the digitally excluded (who are also likely to be older and less digitally literate) (The British Academy, 2022). Online surveys also face difficulties in presenting complex topics, and in minimal control over whether respondents are distracted (Rog and Bickman, 2009). Yet, positively, absence of intersubjective sensitivities helps reduce social desirability bias in the survey, a common problem with ethical and privacy-related research. Moreover, the survey was able to generate a respectable weighted sample of hard-to-reach participants, balanced across gender, socio-economic groups, household income, ethnicity, and UK regions, and covering all ages above 18 years old.

Before data-collection, the research project was approved by the university’s research ethics board, and informed consent from participants was achieved. The survey data was collected in compliance with International Organisation for Standardization (ISO) standards, ISO 20252 (for market, opinion, and social research) and ISO 27001 (for securely managing information assets and data). Anonymized datasets, and full methodological details, are available in the UK Data Archive repository ([DATASET] Laffer, 2023; [DATASET] McStay et al., 2023).

As Stage 2 was shaped by the findings of Stage 1, we report first on Stage 1’s (qualitative) methods and findings, before progressing to Stage 2’s (quantitative) methods and findings.

## 4 Stage 1: focus groups using design fiction and ContraVision

### 4.1 Participants

Across 2021, we conducted 10 focus groups, with 46 participants recruited through a professional research panel. Each focus group typically had four or five participants (to maximize their ability to speak) and lasted less than 2 h (to minimize participant fatigue). As previous studies on attitudes toward emotion profiling in the UK find older adults far less comfortable than younger adults (McStay, 2016; McStay and Rosner, 2021; Bakir et al., 2023b), we purposively sampled participants (Miles et al., 2014) to ensure that age-related differences were well represented, recruiting three younger (18–34 years old) groups (*n* = 12) and three older (+65 years old) groups (*n* = 13). This is a sampling strategy of *maximum* var*iation* to find the widest range of viewpoints. Furthermore, to capture more diverse views, we recruited participants for two groups composed of people who self-identify as disabled (*n* = 10) and two groups belonging to UK ethnic minorities (*n* = 11). These additional groups were important to try to listen to voices that are typically not heard in technology discourses, but that are likely to have unique and important insights. For instance, sociological literature (largely US-focused) highlights how racism (among other things) on data-driven discrimination shapes people’s experiences of data-driven systems (Fisher, 2009; Madden et al., 2017; Benjamin, 2019). Regarding the group that self-identify as disabled, we reasoned that emotion profiling systems could have much to offer disabled people who may, for instance, be more reliant on technology to enable communication and employment, but that emotion profiling technologies can also risk prescribing value-laden benchmarks of what constitutes “normality” (Bakir et al., 2023a). We posited that separate focus groups based on ethnicity and disability might more readily surface unique insights on such factors that could be missed in more general focus groups. However, despite this set-up, in the manipulation theme that we explore in this paper, there was little variation among our various focus group categories.

### 4.2 Deploying design fiction and ContraVision

To help gage attitudes toward complex, abstract, future-facing emotion profiling technologies, our focus groups drew from design fiction principles (Bleecker, 2009; Jensen and Vistisen, 2017) to create a fictional interactive narrative world for our participants, where emotion profiling is situated in everyday circumstances. Participants in each focus group are talked through the interactive narrative by a moderator, where they are presented with a series of different settings involving emotion profiling based on current and likely near-future lived contexts. As well as emotion profiling from social media algorithms and in an emotoy (discussed in this paper), the narrative introduced emotion profiling within a home-hub smart assistant, a digital music service, a bus station surveillance sensor, a sales call evaluation and prompt tool, and a hire-car automated system (the complete design fiction narrative is available at [DATASET] Laffer, 2023; also see Laffer, 2022). Each of these settings introduces the technology and its emotion profiling in terms that are simple (for comprehensibility) and neutral (to minimize social desirability bias). Most settings followed a set sequence. First, a binary choice emerging from the technology use is presented (for example, to accept or reject the emotional AI’s recommendations). This allows participants to make a simple judgment call on initial impressions of the technology, which then prompts group discussion. Second, a “ContraVision” element is introduced with a positive and negative event or outcome for the same scenario, to elicit a wider spectrum of responses than a single presented perspective (Mancini et al., 2010). We took care throughout to avoid utopian or dystopian hyperbole by presenting participants with a reasonably good outcome and a less good outcome. After each of these sequences, participants are asked to reflect on the setting’s emotion profiling and its harms and benefits. The narrative then moves onto the next setting.

Throughout the focus groups, we were not specifically eliciting views on manipulation, or on whether people believed the content that they were exposed to via emotion profiling. Rather our focus was on exploring general views on emotion profiling. For instance, in focus group E1, as the social media setting was introduced, the moderator’s line of questioning included the following to steer conversation, as the narration progressed through this setting:

*So how would we feel if you saw something like this pop up in our newsfeed? … Why do you think you might have seen this? … Are you aware of the sort of profiling and targeting that Facebook does? … What about the added emotional component as well? So if they can profile your likes, your dislikes and how different things make you feel, does that make it worse? … Say if Facebook was collecting more of this kind of emotional data about you so that it could be more accurate in its targeting, would that be a benefit or would we be worried about the data that it was collecting? (Moderator, E1)*

Data coding followed an adaptive approach balancing inductive insights from the data with deductive theory (Layder, 1998). Facilitated by qualitative software package, NVivo, a hand-coded approach was employed to annotate sentences and paragraphs of inductive interest, surfacing data-first codes (Miles et al., 2014) that we abstracted into broader themes, informed by deductive interest in critiques of emotion profiling and manipulation. All authors undertook this process, and then debated and agreed key themes (reported below). Returning to the data, each of these themes was examined to see to what extent they were evident across our participant categories [namely, older (O), younger (Y), disabled people (D), and ethnic minorities (E)] in our social media deepfake and emotoy use cases.

As we discuss below, our focus groups raised concerns about manipulation in two of our various settings: namely, emotion profiling in social media (involving deepfakes, false information and conspiracy theories), and in child oriented emotoys (where the toy responds to the child’s facial and verbal expressions).

#### 4.2.1 Social media use case

**Figure 1:**
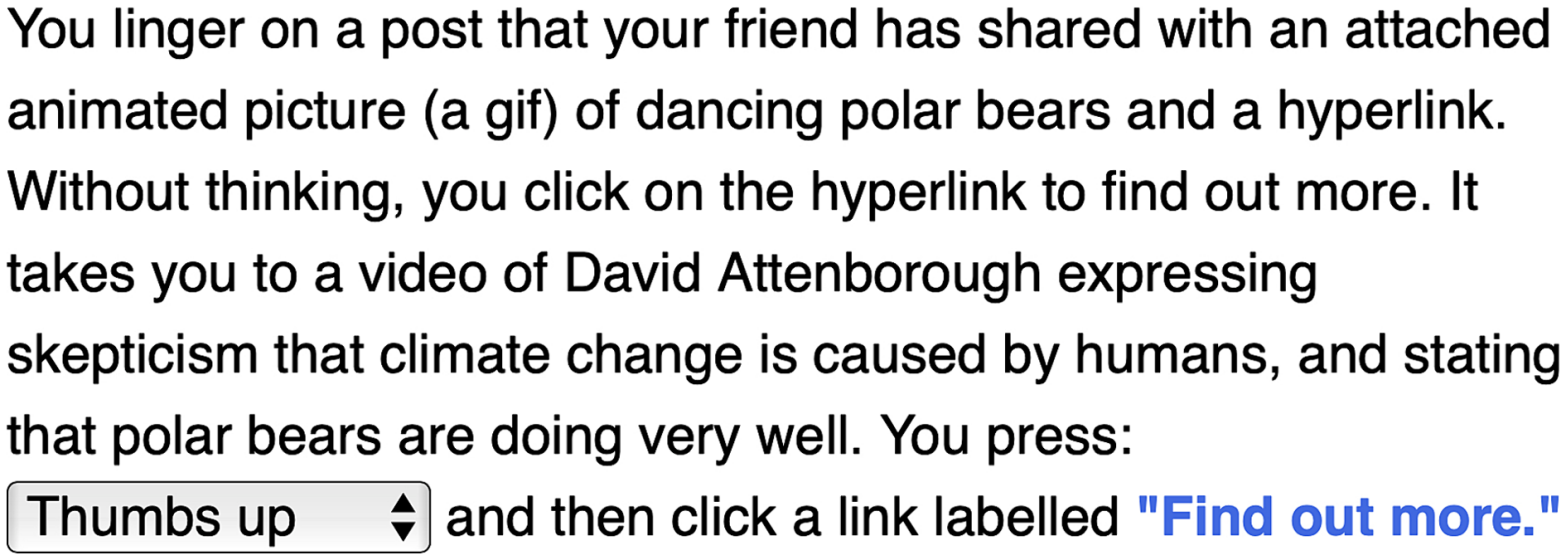
Social media use case: binary choice.

In our design fiction narrative, our social media setting presents emotion profiling from social media algorithms on Facebook. The protagonist scrolls their social media Feed on their mobile phone, encounters emotive content shared by a friend, and is presented with a binary choice (to click thumbs up or thumbs down) on a video of well-known natural historian broadcaster and climate authority, David Attenborough (see Figure 1).

Emotionally baited by the dancing bear animation, the content clicked upon is a deepfake of Attenborough (its deepfake status is discussed only after participants have made their binary choice). Deepfakes are AI-enabled synthetic media that present snippets of audio or video of a person in which their voice, face or body has been digitally altered enabling realistic voice imitation and face swapping. They are becoming easier to produce (Langguth et al., 2021; Gregory, 2023) and are hard for people to distinguish, as shown in studies in the USA, UK, and Netherlands (Dobber et al., 2020; Vaccari and Chadwick, 2020; Köbis et al., 2021; Nightingale and Farid, 2022). Furthermore, they have potential for emotion-driven virality (Bakir and McStay, 2022); have potential to create false memories (Liv and Greenbaum, 2020); and, when combined with AI/machine learning tools, could be tailored to target users with interactive personalized content in social media and in emerging communication formats such as virtual and augmented reality (Gregory, 2023). Together, these factors make deepfakes an important area to study in the realm of emotion profiling and manipulation.

**Figure 2:**
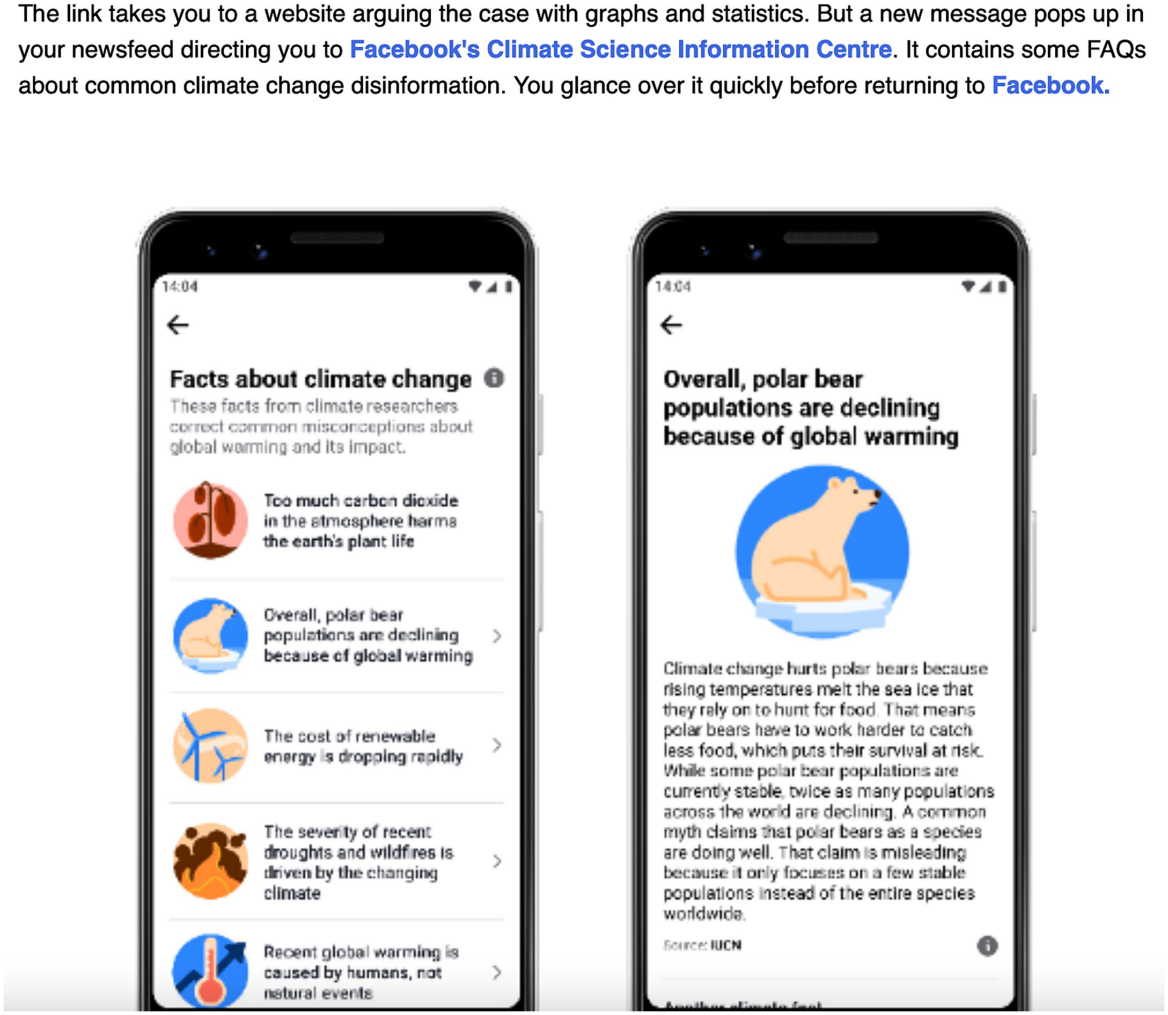
Social media use case: positive ContraVision.

After discussing their binary choices with our participants, the narrative continues with two realistic ContraVision examples (to demonstrate outcomes of clicks and choices, and to stimulate discussion on emotion profiling). The positive ContraVision example redirects the protagonist to Facebook’s Climate Science Information Centre with Frequently Asked Questions about common climate change disinformation (a service that Facebook has operated since 2021 in the UK and 15 other nations to correct virally false and harmful information about climate change). This includes a correction of the false information that polar bears are not impacted by climate change (see Figure 2).

**Figure 3:**
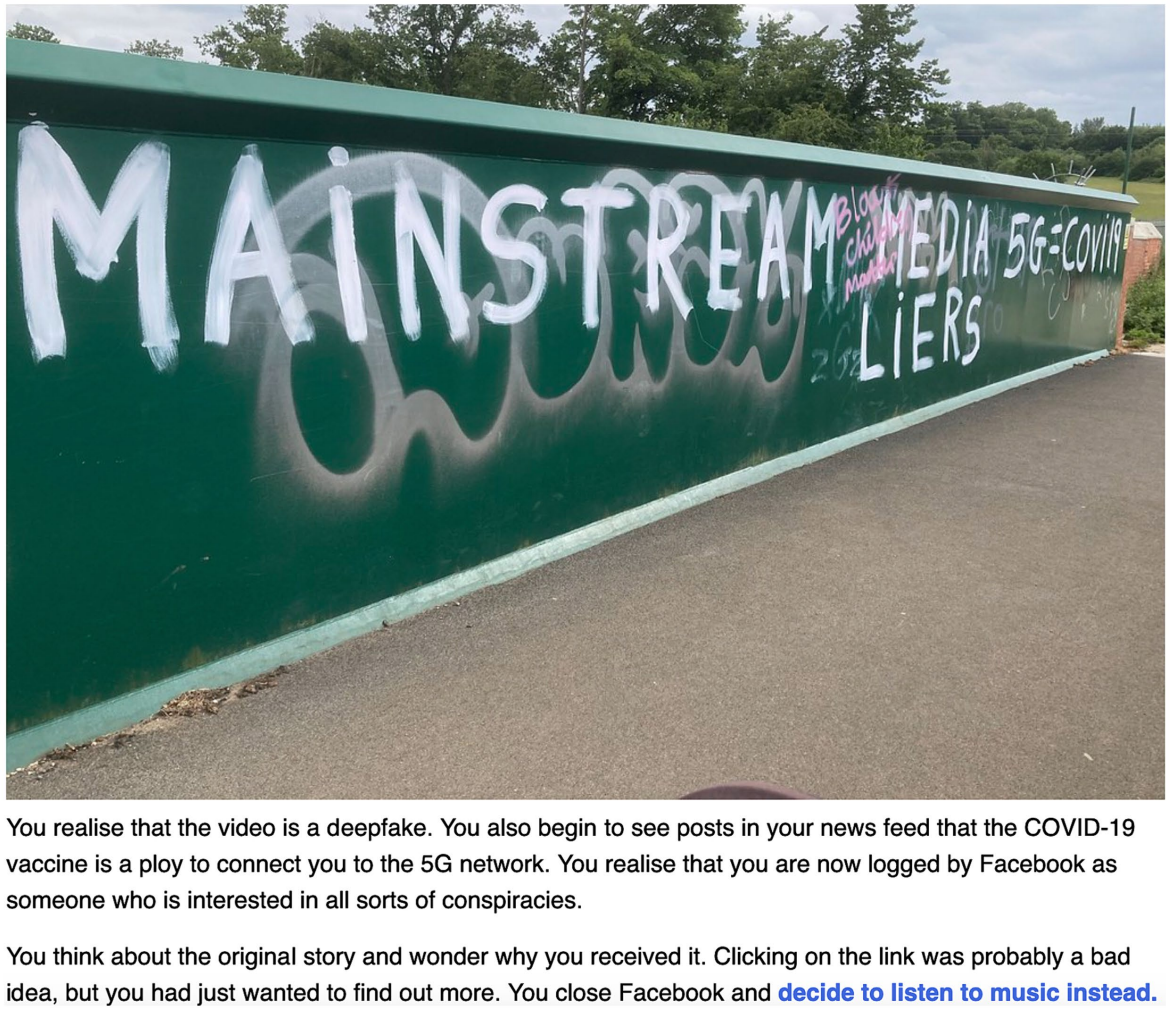
Social media use case: negative ContraVision.

Also realistic, and forming our negative ContraVision example, the protagonist is then bombarded with other conspiracy theories on Facebook because of its algorithms that seek to maximize and monetize user engagement (see Figure 3) [being “interested in pseudoscience” (Sankin, 2020) and in “vaccine controversies” (Wong, 2019) were two of the many ways that Facebook categorized its users to target them with ads across 2019 and 2020]. To exemplify this in the narrative, we chose the then common conspiracy theory of the COVID-19 vaccine being a ploy to connect people to the 5G network.

#### 4.2.2 Emotoy use case

**Figure 4:**
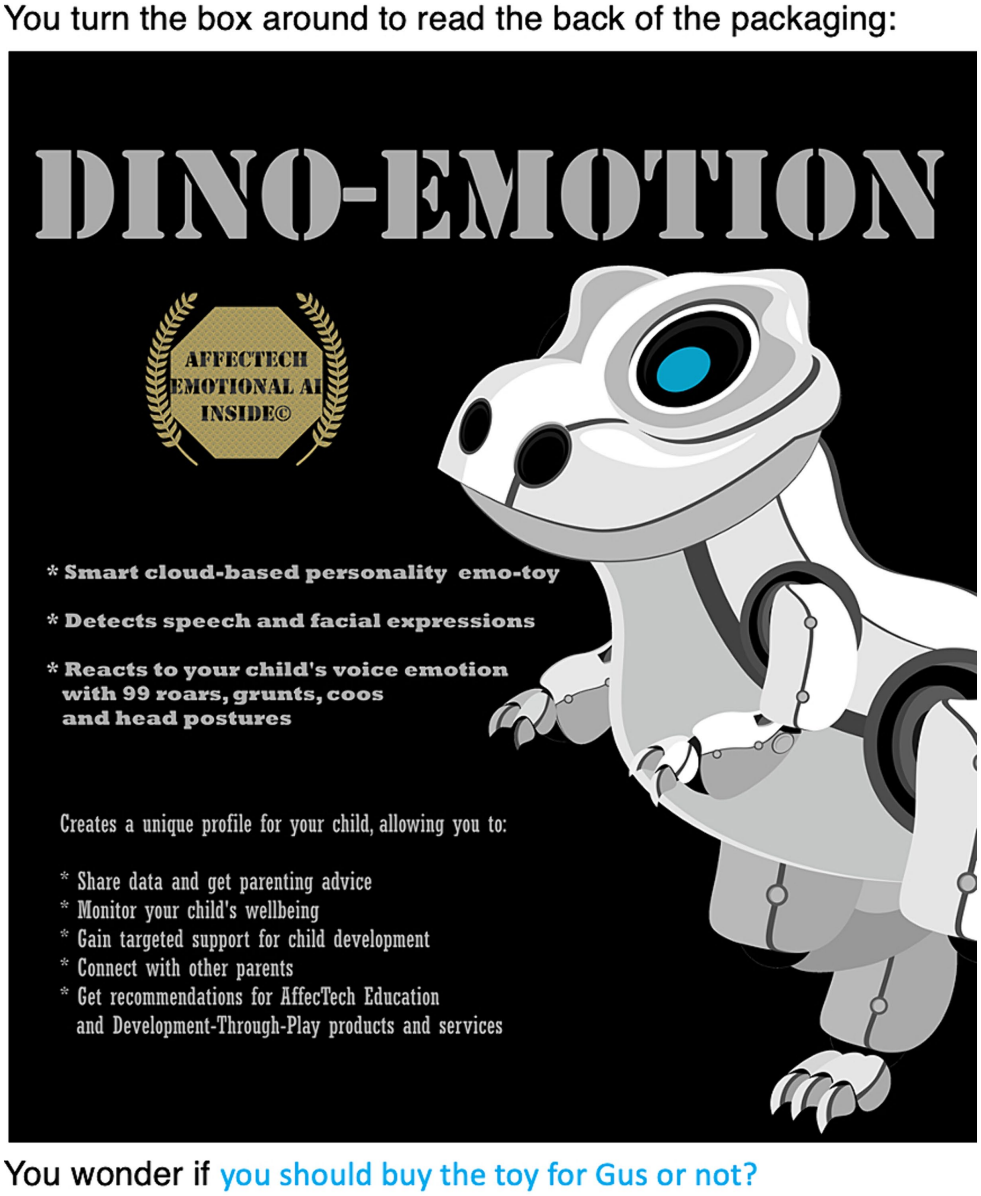
Emotoy use case: binary choice.

For our participants, the children’s toy sector is the second prime site of concern about manipulation from emotion profiling. This second use case involves a “Dino-emotion toy” produced by fictional company, AffecTech, that the protagonist is considering buying as a present for their friend’s child, Gus. Examining the packaging, the protagonist learns that the emotoy “Detects speech and facial expressions” and “reacts to your child’s voice emotion with 99 roars, grunts, coos and head postures” (see Figure 4). Its benefits, presented on the packaging, including the ability to:

*create a unique profile for your child allowing you to: share data and get parenting advice, monitor your child’s well-being, gain targeted support for child development, connect with other parents, get recommendations for AffecTech Education, and Development-through-Play products and services.*

**Figure 5:**
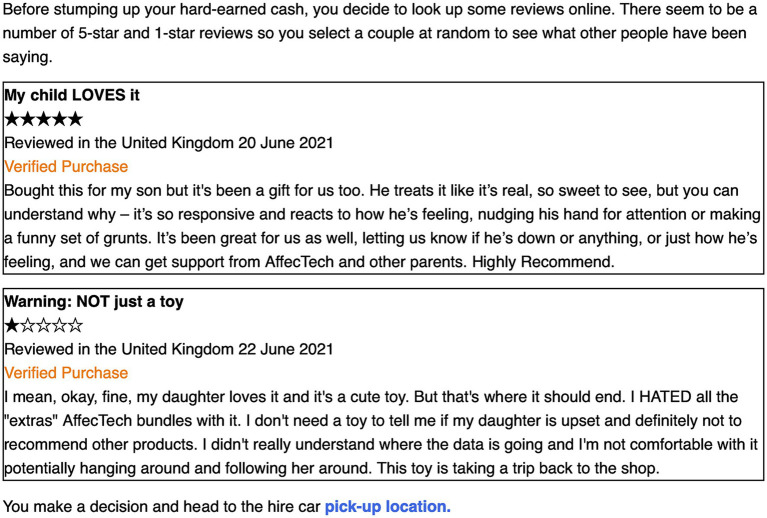
Emotoy use case: positive and negative ContraVisions.

Wondering whether or not to buy the toy (the binary choice—see Figure 4), the protagonist consults online reviews, finding a one-star review expressing concern about where the data is going and discomfort with the data lifecycle (this is the negative ContraVision); and a five-star review, expressing how responsive and funny it is, and that it helps them know their child’s feelings and to get support (the positive ContraVision) (see Figure 5).

### 4.3 Findings from stage 1

Participants offered positive views on emotion profiling, as well as concerns about its potential for manipulation, as we discuss below.

#### 4.3.1 Theme 1—positive feelings on emotion profiling because of fun, relevance and helpfulness

Participants across our four categories appreciate that emotion profiling makes content more fun, relevant, or helpful. For instance, on *fun*, in terms of being emotionally baited by the dancing bear animation in the social media setting, Theo (E2) states: “Well, I’ll be laughing. To see polar bears dancing, it kind of makes me happy. If it’s been shared, I’m assuming it’s being shared by someone that’s a friend.” In the emotoy setting, Brenda (D1) notes, “kids would love to get the reaction back from the toy.” On *relevance*, in the social media setting, Emily (D2) states, “I do like that Facebook does that [serve emotionally profiled content] in another way, because of the advertising”; and Bina (E1) concludes, “It’s not really harmful and it’s relevant and it’s not really intrusive, so it’s all right.” People also appreciate that the emotoy could be *helpful* for parents and for child development. Samuel (Y1) shares: “there’s times when parents are really busy and, yeah, I think this would be a decent substitute.” Isaac (O3) observes: “the feedback that AffecTech education could get, could be used to improve in other areas. So as much as you, the parent, are getting that information to support your child development, it can also be used to improve on things that other children could also access.”

#### 4.3.2 Theme 2—negative feelings on emotion profiling because of manipulation

We were careful to avoid dystopian framings in our design fiction, but a prominent reaction across all our participant categories was dislike of algorithmically profiled emotions, even if it made content more useful. For instance, Theo (E2), aware of how emotion profiling works (from his past employment), sees its *potential for manipulation*:

*It's a human emotion. We own it. It's not supposed to be manipulated by someone else. It's not supposed to be controlled by someone else. Unfortunately, artificial intelligence has given them that opportunity to do that.*

Older, younger, and disabled participants express concern about how such profiling on social media may be *harmful to vulnerable others*, variously affecting their *mental states* and *beliefs*, but also provoking *irrational behavior that goes against their best interests*. Joanne (O2) states:

*I know somebody that does this a lot. And they're a bag of nerves because they are worried about so many things, and that person used to send them to me and I asked them to stop. And I said to them, ‘Why are you doing that?’ Because they know some of it's deepfake but it's like they've [created] a magnet to conspiracies.*

Knowing that it’s deepfake but still passing it on, is clearly irrational, in Joanne’s view. Also highlighting the impact on what she views as irrational anti-vax behavior, Alice (Y3) is *baffled* that her aunt (a nurse):

*has now refused to have her COVID vaccine, because she's reading this stuff on Facebook. … She won't let my cousins have it, she won't let my cousin's children have it done. And I think ‘If you'd never read this on Facebook, everyone would be vaccinated by now.’ And it baffles me how even people who are trained like that can succumb to it and it's just so dangerous.*

Similarly, on *causing irrational behavior*, Lauren (D2) observes that people can:

*end up somewhere, doing things they didn't want to do because of the belief system that they've gone into by reading something that was fake news to start off with. But that's completely blown up all over the internet.*

Beyond harm to vulnerable others, some participants (from the younger groups) even admit to such emotion profiling having *harmful effects on the self* in presenting false or unbalanced content in a *convincing way*. In relation to convincing but false content on COVID-19 vaccines, Niamh (Y1) states: “It’s very dangerous in the wrong hands… Even if you are well-informed, sometimes they are extremely *convincing*. I’ve done it before, and I’ve had to go away and Google it, find information elsewhere to see if it is true.”

Carol (Y3) highlights the problem of repeating profiled, unbalanced, convincing content:

*it's really, really bad to profile people … You just read stories from the people that you think are good, and you don't get other stories and stuff. I'm a victim of this as well, where I've read something on Facebook mindlessly, I didn't even think about it, and then I caught myself saying it, and it was just even like a headline.*

Her phrase “then I caught myself saying it” again highlights the capacity of such convincing content to bypass one’s own rational thought processes, at least temporarily. For both Niamh and Carol, the manipulation seems to be temporary, in that they state that they were able to take corrective action (in seeking other sources, and in becoming self-aware of their own *mindless* behavior).

Participants across all four of our participant categories also express strong concerns about potential for manipulation from the emotoy’s emotion profiling. Older participants were particularly alert to the potential to manipulate parents. For instance, noting the *parent’s vulnerability to commercial manipulation*, Ron (O1) asks:

*Isn't the toy designed to pick up on little, what they would perhaps consider as adverse reactions from the child, in order that they can then push another product? So if they can identify that the child appears to be struggling with the reading or stuttering over certain words, or having some other reaction. If they've got a product that fits that, then that's exactly what they're looking for. That's the whole point of it.*

Expressing concerns about the *parent’s vulnerability to psychological pressure*, Linda (O3) states, “I just think, it could put pressure on the parents, because if a toy’s reporting back that your child needs more this, that and the other, then you’d feel a bad parent then, would not you?” Noting another aspect of the *parent’s vulnerability to psychological pressure*, Phillip (O2) observes.

*There's a couple of phrases there, monitoring your child's wellbeing and targeted support for child development, suggest to me, it's playing for the hypochondriac parent, you know, ‘my child isn't able to do this at this age. My God, there's something wrong.’ That sort of thing, which I could see could generate quite a lot of angst.*

Others express *concerns about where the data goes and who might exploit it*. For instance, Paul (Y2) thinks, “it just depends on what the data is used for, does not it? If the data is used to promote other toys, target ads, then I think there’s an issue.” Expressing concerns about the emotoy’s advice to parents constituting *unwanted interference,* Ruby (D1) retorts, “this bit where it says, ‘get parenting advice,’ what, hearing this advice from a toy? I’ve managed to raise them for the last 9 years. I think I’m okay, thank you. I do not like that bit at all.” In terms of fears that the *AI (rather than the parent or child) has control,* Bina (E1) explains, “we do not know the AI behind it and everything, it might try to correct your kid in a path that is right for the AI, but not according to your values that you want to teach your child.”

## 5 Stage 2: national survey

### 5.1 Method

Our national survey asks closed-ended questions about 10 use cases, variously reflecting issues in studies on emotional AI, studies on manipulation, and themes emerging from our prior focus groups. As the social media deepfake and emotoys settings in the focus groups raised strong concerns about manipulation, we ensured that our survey had two analogous use cases: one where digital manipulation tools (deepfakes and social media bots) are used to increase the emotional power and spread of messages on social media; and another where an emotoy reacts to the child’s biometrically gleaned emotions. These two use cases were presented alongside eight other use cases on emotion profiling (covering elderly care homes, policing, private security, schools, employment, cars, home robots, and political campaigning) (although not discussed here as these other use cases did not seek views on manipulation, we report on them at [DATASET] McStay et al., 2023).

The survey introduces the overall topic of emotion profiling with the phrase: “We would now like to ask your opinion on use of technologies that try to measure and understand emotions (e.g., through computer analysis of social media posts, facial expression, voice, heart rate, gesture, and other data about the body).” Each of our 10 use cases is then presented neutrally followed by three to five closed-ended questions exploring themes of most relevance to each use case (the same themes were not tested across all 10 use cases, to minimize survey fatigue). By grounding each question in an applied use case, and by scrutinizing each question for clarity, we ensured that our questions were readily understandable. For each use case, we ensured that both positive and negative themes are explored via the closed-ended questions.

The digital manipulation tools use case was presented as follows:

*Artificial intelligence tools can generate realistic audio-visual duplicates of people doing or saying things that they never actually did or said (so called 'deepfakes'). These can be used to deliver emotionally powerful but false messages, attributed to politicians, leaders and celebrities. Computer programs can also seem like real human users ('bots') and can be used to amplify messages on social media, often in favor of, or against a political or social issue. Please state how strongly you agree or disagree with the following statements about what you have just read.*

*1. I would be comfortable for these forms of digital manipulation tools to be used to promote political and social causes.*

*2. I would have concerns if these digital manipulation tools were used because it will make it harder for me to know what messages or people are real or fake.*

*3. I would have concerns if these digital manipulation tools were used because it will make it harder for me to know what messages or people are really popular or if they are being artificially amplified.*

In this use case, we introduced two digital manipulation tools—deepfakes (a novel tool at the time of the focus groups) and social media bots (a more common tool). Both have potential for emotion-driven virality. Bots are often used in influence campaigns to amplify marginal voices and ideas by inflating the number of engagements they receive, to impersonate real people, and to flood social media with information (Schneier, 2020) (with emotional and false content being more viral, Vosoughi et al., 2018; Goldenberg and Gross, 2020). Deepfakes can elicit more visceral, emotional, and empathic responses than text-based media (Ajder and Glick, 2021; Langguth et al., 2021); and while often critiqued for propagating false information, they can also be used for pro-social campaigns. For instance, Pakistani climate-change initiative, Apologia Project, depicts current world leaders apologizing from the year 2032 for their previous inaction on environmental crises, with its rhetorical power deriving from the leaders’ sincere remorse and knowledge that they could have done more (Ajder and Glick, 2021).

This use case’s closed-ended responses include a positive evaluation of such a use case for the purposes of promoting political and social causes (response 1). Response 1 builds on our focus groups’ positive theme of being comfortable with receiving relevant, emotionally profiled promotional content on social media, as well as studies that point to the creative potential of deepfakes in campaigning (Ajder and Glick, 2021). Responses 2 and 3 ask about concerns about being able to tell what is real or authentic—a common problem with deepfakes and bots (Dobber et al., 2020; Vaccari and Chadwick, 2020; Köbis et al., 2021; Nightingale and Farid, 2022). We chose to phrase these concerns as being about the self, as our focus group sub-theme of *harmful effects on the self* shows some participants openly admitting to being confused or influenced by emotionally profiled, false content.

Our second use case, on emotoys, is phrased as follows:

*This question is about interactive toys for children up to 12 years old. Toymakers are interested in building toys with capabilities for basic conversations, meaning they can increasingly understand and derive meaning from children's speech. These toys would also try to interpret emotion in child speech, through tone of voice, so that the toy can respond appropriately by adapting play activities or trying to cheer them up if they are sad. Please state how strongly you agree or disagree with the following statements about what you have just read.*

*1. I would be comfortable with this as it sounds like fun. I wish I had toys like this when I was younger.*

*2. I would have concerns about what the toy is saying to the child, how it is handling conversation with the child, and maybe even what it is advising the child to do or think.*

*3. I would have concerns about where the emotion data about conversations would go and who could access it, e.g. advertisers trying to sell the child more toys.*

The closed-ended responses include a positive evaluation of such a use case as being *fun* (response 1), and negative evaluations expressing concerns about what the emotoy would say or *advise the child* (response 2), and about *where the data goes* and arising potential for *commercial manipulation* (response 3). These responses, positive and negative, are directly informed by themes from our focus groups.

### 5.2 Survey results

For most of our survey’s 10 use cases, a majority of UK adults are uncomfortable with the emotional AI (see [DATASET] McStay et al., 2023). People are least comfortable with the use case where digital manipulation tools (deepfakes and social media bots) are used to increase the emotional charge of a message (through seemingly authentic messages or through perceived popularity) to promote political and social causes: only 19% are comfortable while 59% are uncomfortable. Large majorities (nearly three quarters) think that such tools will make it harder to know what messages or people are real or fake or are genuinely popular or artificially amplified (see Table 1).

**Table 1:** UK adults’ level of comfort with emotional AI in digital manipulation tools (deepfakes and social media bots).

**Use case**	**Comfortable**	**Do not know**	**Uncomfortable**
**Positive: for general good or social use**			
I would be comfortable for these forms of digital manipulation tools to be used to promote political and social causes	19%	22%	59%
**Negative: manipulation**			
I would have concerns if these digital manipulation tools were used because it will make it harder for me to know what messages or people are real or fake	9%	19%	72%
I would have concerns if these digital manipulation tools were used because it will make it harder for me to know what messages or people are really popular or if they are being artificially amplified	7%	21%	73%

Source: UK survey, *n* = 2,068 UK adults conducted by ICM Unlimited across 29 June–1 July 2022.

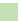
 >66% comfortable or <34% uncomfortable, 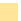 34–66% comfortable or uncomfortable, 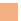 0–33% comfortable or >66% uncomfortable.

With emotoys, people are divided or unsure about the use case of emotional AI helping toys respond to the child appropriately for fun (38% are comfortable, 34% uncomfortable and 28% do not know). However, over two thirds are uncomfortable with where the emotion data goes and how it may be used to commercially manipulate the child (68% uncomfortable). Similarly, 63% express concerns about what the toy is saying to child, how it is handling conversation with child, and what it is advising the child to do or think (see Table 2).

**Table 2:** UK adults’ level of comfort with emotional AI in toys.

**Use case**	**Comfortable**	**Do not know**	**Uncomfortable**
**Positive: for general good or social use**			
I would be comfortable with this as it sounds like fun. I wish I had toys like this when I was younger	38%	28%	34%
**Negative: manipulation**			
I would have concerns about what the toy is saying to the child, how it is handling conversation with the child, and maybe even what it is advising the child to do or think	13%	24%	63%
I would have concerns about where the emotion data about conversations would go and who could access it, e.g., advertisers trying to sell the child more toys	9%	22%	68%

Source: UK survey, *n* = 2,068 UK adults conducted by ICM Unlimited across 29 June–1 July 2022.

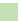
 >66% comfortable or <34% uncomfortable, 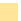 34–66% comfortable or uncomfortable, 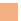 0–33% comfortable or >66% uncomfortable.

We find that age makes the largest difference to levels of comfort or concern with these different use cases (with no strong, clear signals according to other demographic markers). Table 3 shows that young adults are more comfortable with use of digital manipulation tools to increase the emotional amplification of a message to promote political and social causes, although the percentage is far from constituting a majority of young adults. In this use case, older adults are more concerned than younger adults about the potential for confusion about what is real or popular. Similar age-related patterns are found with emotoys (with the exception of the concern about advice proffered by the emotoy, where most of the youngest and oldest groups are concerned, but with a dip in concern among 45–54-year-olds) (see Table 4).

**Table 3:** Age differences in UK adults’ levels of comfort with emotional AI in digital manipulation tools (deepfakes and social media bots).

**Use case**	**Total**	**18–24 years**	**25–34 years**	**35–44 years**	**45–54 years**	**55–64 years**	**65–74 years**	**75+ years**
**Digital manipulation tools**—I would be comfortable for these forms of digital manipulation tools to be used to promote political and social causes	19%	29%	34%	24%	17%	10%	8%	5%
**Digital manipulation tools**—I would have concerns if these digital manipulation tools were used because it will make it harder for me to know what messages or people are real or fake	72%	63%	72%	64%	72%	75%	77%	84%
**Digital manipulation tools**—I would have concerns if these digital manipulation tools were used because it will make it harder for me to know what messages or people are really popular or if they are being artificially amplified	73%	64%	72%	60%	72%	78%	78%	90%

Source: UK survey, *n* = 2,068 UK adults conducted by ICM Unlimited across 29 June–1 July 2022.

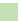
>66% comfortable or <34% uncomfortable, 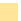 34–66% comfortable or uncomfortable, 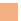 0–33% comfortable or >66% uncomfortable.

**Table 4:** Age differences in UK adults’ levels of comfort with emotional AI in toys.

**Use case**	**Total**	**18–24 years**	**25–34 years**	**35–44 years**	**45–54 years**	**55–64 years**	**65–74 years**	**75+ years**
**Toys**—I would be comfortable with this as it sounds like fun. I wish I had toys like this when I was younger	38%	44%	48%	41%	37%	30%	31%	31%
**Toys**—I would have concerns about what the toy is saying to the child, how it is handling conversation with the child, and maybe even what it is advising the child to do or think	63%	68%	64%	63%	51%	65%	69%	68%
**Toys**—I would have concerns about where the emotion data about conversations would go and who could access it, e.g., advertisers trying to sell the child more toys	68%	67%	68%	65%	60%	70%	76%	79%

Source: UK survey, *n* = 2,068 UK adults conducted by ICM Unlimited across 29 June–1 July 2022.

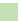
 >66% comfortable or <34% uncomfortable, 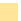 34–66% comfortable or uncomfortable, 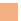 0–33% comfortable or >66% uncomfortable.

## 6 Discussion

With about five decades of research on the sociology of emotions (Hochschild, 1975; Bericat, 2016), understanding is improving regarding the social nature of emotions, the emotional nature of social reality, how emotions may frame what we see, the complexities of defining emotion, the relationship between physiology and society (and culture), and ethical questions about neoliberal interests in defining these answers (Ahmed, 2010; Davies, 2015; McStay, 2018; Stark and Hoey, 2020). The global technology industry, for example, has a vested interest in simplistic accounts of emotional life, due to the global scale they operate at. Connected, other vested interests are accounts of emotion that accord with object and facial recognition (despite being staffed by individuals and teams well-aware that these technologies are, at best, highly simplistic). For emotional AI interests, the sociology of emotion intersects with the political economy of AI technologies, particularly in regard to how emotion is prescribed and defined (McStay, 2018, 2023; Stark and Hoey, 2020). Yet, there has been a disconnect between the growing multi-disciplinary literatures on emotion profiling by the technology industry and on manipulation, and there is a paucity of research on people’s views on the use of emotion profiling to potentially influence them—a lacuna we have attempted to address in this paper.

### 6.1 Discussion of qualitative findings

Despite purposively sampling for diverse views, we find points of convergence across all our participant categories as two of our design fiction’s emotional AI settings (a social media deepfake and an emotoy) strongly raised concerns about *manipulation* (while also noting the benefits of *fun*, *relevance* and *helpfulness*). As we explain below, our qualitative study finds concerns about two forms of manipulation in play: (1) where emotion profiling is used covertly to exploit users’ cognitive or affective weaknesses and vulnerabilities (this found in both the social media and emotoys settings); and (2) where emotion profiling damages people’s capacity for rational thought and action (this found only in the social media setting).

Discussing the emotoys setting first, our study finds much concern that emotion profiling may be used covertly to exploit users’ cognitive or affective weaknesses and vulnerabilities. This includes concerns about the *parent’s vulnerability to commercial manipulation* (Ron’s account), and *to psychological pressure* (Linda’s and Phillip’s accounts). These reflect concerns raised by a study into the views of experts on emotional AI, data ethics and the toy industry (McStay and Rosner, 2021), as well as fears found in recent UK-based nationally representative surveys into people’s attitudes toward a wide variety of AI use cases, discussed earlier (Centre for Data Ethics and Innovation, 2022; Ada Lovelace Institute and The Alan Turing Institute, 2023). Also of note is that the emotoy’s advice to parents is seen as constituting *unwanted interference* (Ruby’s account) as well as raising fears that the *AI (rather than the parent or child) has control* (Bina’s account). This adds nuance to previous surveys that find that people are more negative about AI where it is seen as replacing (rather than augmenting) human decision-making (Centre for Data Ethics and Innovation, 2022; Ada Lovelace Institute and The Alan Turing Institute, 2023; Luminate, 2023). The *unwanted interference* element indicates that, at least for some in our focus groups, it is not a question of whether AI augments rather than replaces humans, but rather that there is no desired role for AI at all in parent–child relationships.

In our social media deepfake setting, our focus groups again raise concerns that emotion profiling may be used covertly to exploit users’ cognitive or affective weaknesses and vulnerabilities. Participants are concerned that emotion profiling is *harmful to unknown and known vulnerable others* in *negatively* affecting *mental states* (anxiety, in Joanne’s account about sharing deepfakes) as well as *beliefs* (Lauren’s account). Our participants’ fears that emotion profiling and its targeting of false information negatively affects people’s mental states is in tune with past research that finds strongly negatively emotionalised environments around false information (Vosoughi et al., 2018; Barfar, 2019; Paschen, 2019). Our participants’ fears about the manipulability of others’ beliefs are supported by studies that show that emotionalised online environments influence people’s real-world attitudes on polarization, politics, or belief in false information (Martel et al., 2020; Walter et al., 2020; Van Bavel et al., 2021), although as Section 2.2 shows, other studies show minimal influence on these real-world attitudes in political contexts (Nyhan et al., 2023; Guess et al., 2023a,b). Some younger participants also find emotion profiling to have *harmful effects on the self* in presenting false or unbalanced content in a *convincing* way, although they also indicate that the manipulation is temporary, as they were able to correct their *confusion* or *mindless* action (Niamh’s and Carol’s accounts). Their confusion, even if just temporary, accords with a national survey into UK adults’ ability to tell if a deepfake is true or false, where a third of respondents were uncertain if is true or false and 16% were deceived (Vaccari and Chadwick, 2020).

Perhaps our most interesting qualitative insight from the social media use case is that participants are concerned that emotion profiling provokes *irrational behavior that goes against people’s best interests*. This threads through accounts from Joanne, Alice, Lauren, and Carol where we are told about anxious people who pass on deepfakes even when they know them to be false; who have anti-vax behaviors despite being trained nurses; who do things they did not want to do by being sucked into belief systems that they were introduced to by fake news; and by catching themselves repeating profiled, unbalanced, convincing content that they had been exposed to online. This expands our understanding of how manipulation may occur. Section 2 defined manipulation as “undermining of autonomy, or freedom to choose, reflect and deliberate.” It outlined that this may happen by providing us with *insufficient information* and by *deceiving*, *coercing,* or *incentivizing* us (Bakir et al., 2019); by exploiting our *cognitive or affective weaknesses* (Susser et al., 2019); or by side-stepping our *capacity for reflection and deliberation* (Sunstein, 2016). To this list we add that manipulation may occur by *damaging our very capacity for rational thought and action.*

### 6.2 Discussion of quantitative findings

Our survey took care to present neutrally our two emotion profiling use cases that broached aspects of manipulation, and to present positive as well as negative statements for participants to express views on. Nonetheless, large majorities express concern about the potential for manipulation in these two use cases. In our first use case, namely, digital manipulation tools to increase the emotional power and spread of messages on social media, we find that 59% are uncomfortable with digital manipulation tools (deepfakes and social media bots) being used to promote political and social causes (Table 1). Even more are concerned about the potential for these tools to cause confusion about what is real (either through seemingly authentic messaging or through perceived popularity); nearly three quarters think that such tools will make it harder to know what messages or people are real or fake or are genuinely popular or artificially amplified. This accords with studies that show that people are poor at recognizing deepfakes and false content online (Dobber et al., 2020; Vaccari and Chadwick, 2020; Köbis et al., 2021; Nightingale and Farid, 2022; Vaccari et al., 2022).

In our second use case (voice-based emotoys that respond appropriately to the child’s speech and tone of voice), our survey finds that people are divided or unsure about whether this is a fun prospect (Table 2). Far more (about two thirds) are uncomfortable with where the emotion data goes (this echoing concerns found in McStay and Rosner’s (2021) UK-based survey of parents). About two thirds of our survey’s participants are also uncomfortable with how the emotion data may be used to manipulate the child, whether in terms of commercial manipulation or mental and behavioral manipulation. This accords with high-level fundamental rights for children. The United Nations Convention on the Rights of the Child recognizes that children have the right to have their privacy protected (article 16), that their lives should not be subject to excessive interference, and that regardless of cultural context a child should be able to have their own ideas, thoughts, opinions, and beliefs (UNCRC, 2013; McStay, 2023).

Our survey also finds that the biggest variances in levels of comfort or concern with these different use cases is found in age, with older adults more concerned and younger adults more comfortable (Tables 3, 4). This finding accords with past surveys into UK attitudes toward emotional AI (McStay, 2016; McStay and Rosner, 2021; Bakir et al., 2023b). Why older people are more concerned is not clear from past studies, but it is instructive that our focus groups (the emotoys use case) find older participants being particularly alert to the potential of such emotion profiling to manipulate parents. This perhaps stems from older participants’ experiential sensitivity to the vulnerability of parents, as well as appreciation of the absence of such technologies when they were parents themselves (although this is not specifically articulated by our older participants).

### 6.3 Governance implications

As politicians and policymakers across the world seek to establish whether and how to regulate AI to build public trust in these technologies, our findings are instructive. Our national survey on UK adults’ attitudes toward emotional AI in use cases that have potential for manipulation finds only small minorities seeing the positive aspects of such use cases. It finds widespread concern about their potential for manipulation, this reflecting concerns in multidisciplinary academic scholarship, although empirical evidence of actual impact is so far lacking or unclear (as detailed in Section 2).

Indeed, the UK public’s views are in accordance with the thrust of the EU’s draft AI Act that strongly asserts the need to protect human autonomy and to ensure that human behavior is not harmfully distorted by AI. This is affirmed by all emotion recognition technologies and some uses of social media (for instance in political campaigning) being given “high risk” status (if they are not prohibited outright), requiring greater regulatory oversight (European Parliament, 2023). With the UK diverging from the EU, and currently seeking to allow existing regulators to flexibly determine what AI use cases are risky and require regulatory attention (Department for Science, Innovation, and Technology and Office for Artificial Intelligence, 2023), this suggests that UK regulators of digital content (Ofcom) and data protection (the Information Commissioners’ Office) should, as a minimum, be highly alert to potentially manipulative uses of emotional AI. Useful foresight work has already been done by the Information Commissioners’ Office (ICO, 2022), that notes scope to surveil subconscious behaviors and responses, so this paper adds empirical heft to these observations.

We recommend regulatory calibration, in line with our paper’s clarification of the two types of manipulation that concern people.

Firstly, where emotion profiling is being used covertly to *exploit users’ cognitive or affective weaknesses and vulnerabilities’*, our findings suggest desire for strong social protections from such applications of emotional AI. We suggest that given these concerns, regulators should not be timid in advancing a proactive and precautionary regulatory stance that would take priority over enabling innovation in AI.

Where emotion profiling is being used to *attack people’s capacity for rational thought and action*, our focus group findings suggest that at least some people may be able to take corrective action (in seeking other sources, and in becoming self-aware of their own behavior). However, with the survey finding large majorities concerned about the potential for digital manipulation tools to cause confusion about what is real or popular, this suggests that interventions that reduce people’s confusion will be necessary. This is unlikely to be as simple as increasing people’s digital literacy skills, as recent research finds that this not only reduces belief in false information, but in all information (Hoes et al., 2023). Furthermore, attempting to improve media literacy skills on false information is complicated, and potentially undermined, by people’s habits or ‘mental shortcuts’ that in turn draw on factors such as their fears, identity expression and ways of reasoning (Liv and Greenbaum, 2020; Ecker et al., 2022). Rather, then, the answer is likely to lie further up the supply chain, by reducing or modulating exposure to this sort of content in the first place. However, content moderation of false or unbalanced information can run afoul of free speech rights (United Nations, 2022), and attracts accusations of censorship, for instance from users with minority beliefs (Strong et al., 2023). Moreover, platform-provided interventions to help users while online (e.g., by providing real-time information on content they are about to view) has been found to annoy UK users, who then increasingly ignore the interventions (Ofcom, 2023). Similarly, labeling of content (such as by third parties) to indicate if it has been manipulated raises questions about the labels’ interpretability and accessibility by different audiences; and rapid, high-quality media forensics analysis of content believed to be AI-generated are not widely available, with the resulting gap between analysis and timely public understanding being easily exploitable by malicious actors (Gregory, 2023). Given these problems, the answer may lie, instead, in paying attention to the act of emotion profiling itself that spreads and targets such content. With this attacking the business models of globally dominant engagement-driven platforms, it will fall upon regulators to encourage, and socially oriented developers to seek, forms of engagement that do not rely on automated emotion profiling.

### 6.4 Limitations and future research

Our survey is limited in that it is possible that respondents to the social media use case may have taken the phrase of “digital manipulation tools” (in the response options) to be more negative than was intended (the term “digital manipulation tools” can refer to a wide range of content alteration tools—and it is this neutral meaning that we intended): this might, in turn affect the results to be more negative than they otherwise might have been. Furthermore, the emotoy response options were multi-barrelled, including a plurality of factors for discomfort that make the results more difficult to interpret than more singular responses. Future survey-based studies should aim for more singular response options.

Our empirical study is limited to the UK context, and to exploring people’s views rather than their practices. As emotional AI becomes increasingly deployed world-wide across multiple sectors, it would be useful to examine people’s actual experiences of, and strategies for resisting, manipulation. Future research could also productively examine to what extent the roll-out of emotional AI embraces or avoids use cases involving manipulative intent and outcomes. Finally, similar studies (on use cases and perceptions) beyond the UK would be illuminating in helping inform commercial developers of emotional AI systems, as well as in the development of wider supra-national legislation, specific national legislation, and application of international rights.

## Data availability statement

The datasets presented in this study can be found in online repositories. The names of the repository/repositories and accession number(s) can be found at: UK Data Service: https://reshare.ukdataservice.ac.uk/855688/ and https://reshare.ukdataservice.ac.uk/856708.

## Ethics statement

The studies involving humans were approved by Bangor University Research Ethics Committee. The studies were conducted in accordance with the local legislation and institutional requirements. The participants provided their written informed consent to participate in this study.

## Author contributions

VB: Conceptualization, Data curation, Formal analysis, Funding acquisition, Investigation, Methodology, Supervision, Writing – original draft, Writing – review & editing, Project administration. AL: Conceptualization, Data curation, Formal analysis, Investigation, Methodology, Visualization, Writing – review & editing. AM: Conceptualization, Data curation, Formal analysis, Funding acquisition, Investigation, Methodology, Project administration, Supervision, Writing – review & editing. DM: Conceptualization, Data curation, Funding acquisition, Methodology, Supervision, Writing – review & editing. LU: Conceptualization, Data curation, Formal analysis, Funding acquisition, Investigation, Methodology, Supervision, Writing – review & editing.

## References

[ref1] Ada Lovelace Institute and The Alan Turing Institute. (2023). How do people feel about AI? A nationally representative survey of public attitudes to artificial intelligence in Britain. Available at: https://www.adalovelaceinstitute.org/report/public-attitudes-ai/ (Accessed November 7, 2023).

[ref2] Ahmed S. (2010). The promise of happiness. Durham: Duke University Press.

[ref3] Ajder H. Glick J. (2021). Just joking! Deepfakes, satire and the politics of synthetic media. WITNESS and MIT open documentary lab. Available at: https://cocreationstudio.mit.edu/just-joking/ (Accessed November 7, 2023).

[ref4] Alegre S. (2017). Opinion. Rethinking freedom of thought for the 21st century. Eur. Hum. Rights Law Rev. 3, 221–233.

[ref5] Alegre S. (2022). Freedom to think: The long struggle to liberate our minds. London: Atlantic Books.

[ref6] Andalibi N. Buss J. (2020). “The human in emotion recognition on social media: attitudes, outcomes, risks”, In: CHI '20: Proceedings of the 2020 CHI Conference on Human Factors in Computing Systems, April 2020, 1–16.

[ref7] ARTICLE 19. (2021). Emotional entanglement: China’s emotion recognition market and its implications for human rights. Available at: www.article19.org/wp-content/uploads/2021/01/ER-Tech-China-Report.pdf (Accessed November 7, 2023).

[ref8] Bakir V. (2020). Psychological operations in digital political campaigns: assessing Cambridge Analytica’s psychographic profiling and targeting. Front. Polit. Commun. 5:67. doi: 10.3389/fcomm.2020.00067

[ref9] Bakir V. Herring E. Miller D. Robinson P. (2019). Organised persuasive communication: a new conceptual framework for research on public relations, propaganda and promotional culture. Crit. Sociol. 45, 311–328. doi: 10.1177/0896920518764586

[ref10] Bakir V. Laffer A. McStay A. (2023a). Human-first, please: assessing citizen views and industrial ambition for emotional AI in recommender systems. Surveil. Soc. 21, 205–222. doi: 10.24908/ss.v21i2.16015

[ref11] Bakir V. Laffer A. McStay A. (2023b). Blurring the moral limits of data markets: biometrics, emotion and data dividends. AI & Soc. doi: 10.1007/s00146-023-01739-5

[ref12] Bakir V. McStay A. (2022). ‘Core incubators of false information online’ in Optimising emotions, incubating falsehoods. Cham: Palgrave Macmillan.

[ref9002] Barassi V. (2020). Child Data Citizen: How Tech Companies Are Profiling Us from before Birth. Cambridge, MA: MIT Press.

[ref13] Barfar A. (2019). Cognitive and affective responses to political disinformation in Facebook. Comput. Hum. Behav. 101, 173–179. doi: 10.1016/j.chb.2019.07.026

[ref14] Barrett L. F. Adolphs R. Marsella S. Martinez A. M. Pollak S. D. (2019). Emotional expressions reconsidered: challenges to inferring emotion from human facial movements. Psychol. Sci. Public Interest 20, 1–68. doi: 10.1177/1529100619832930, PMID: 31313636 PMC6640856

[ref15] Benjamin R. (2019). Race after technology: Abolitionist tools for the new Jim code. Cambridge: Polity.

[ref9003] Bericat E. (2016). The sociology of emotions: Four decades of progress. Current Sociology, 64, 491–513. doi: 10.1177/0011392115588355

[ref16] Bleecker J. (2009). Design fiction: a short essay on design, science, fact and fiction. Near future laboratory. Available at: https://blog.nearfuturelaboratory.com/2009/03/17/design-fiction-a-short-essay-on-design-science-fact-and-fiction/ (Accessed November 7, 2023).

[ref17] Centre for Data Ethics and Innovation (2022). Public attitudes to data and AI: tracker survey (wave 2). Available at: https://www.gov.uk/government/publications/public-attitudes-to-data-and-ai-tracker-survey-wave-2 (Accessed November 7, 2023).

[ref18] Corbu N. Bârgăoanu A. Durach F. Udrea G. (2021). Fake news going viral: the mediating effect of negative emotions. Media Literacy and Academic Research 4, 58–85. Available at: https://www.mlar.sk/wp-content/uploads/2021/12/4_Corbu.pdf

[ref19] Council of the EU (2022). Digital services act: Council and European Parliament provisional agreement for making the internet a safer space for European citizens. Available at: https://www.consilium.europa.eu/en/press/press-releases/2022/04/23/digital-services-act-council-and-european-parliament-reach-deal-on-a-safer-online-space/ (Accessed November 7, 2023).

[ref20] Davies W. (2015). The happiness industry: How the Government & big Business Sold us wellbeing. London: Verso.

[ref21] Department for Science, Innovation, and Technology and Office for Artificial Intelligence (2023). A pro-innovation approach to AI regulation. Available at: https://www.gov.uk/government/publications/ai-regulation-a-pro-innovation-approach/white-paper (Accessed November 7, 2023).

[ref22] Dobber T. Metoui N. Trilling D. Helberger N. de Vreese C. (2020). Do (microtargeted) deepfakes have real effects on political attitudes? Int. J. Press/Politics 26, 69–91. doi: 10.1177/1940161220944364

[ref23] Ecker U. K. H. Lewandowsky S. Cook J. Schmid P. Fazio L. K. Brashier N.. (2022). The psychological drivers of misinformation belief and its resistance to correction. Nat. Rev. Psychol. 1, 13–29. doi: 10.1038/s44159-021-00006-y

[ref24] European Commission (2021). Proposal for a regulation of the European Parliament and of the council laying down harmonised rules on artificial intelligence (artificial intelligence act) and amending certain union legislative acts. Brussels, COM(2021) 206 final 2021/0106 (COD). Available at: https://digital-strategy.ec.europa.eu/en/library/proposal-regulation-laying-down-harmonised-rules-artificial-intelligence (Accessed November 7, 2023).

[ref25] European Parliament (2023). Amendments adopted by the European Parliament on 14 June 2023 on the proposal for a regulation of the European Parliament and of the council on laying down harmonised rules on artificial intelligence (artificial intelligence act) and amending certain union legislative acts. COM(2021)0206 – C9-0146/2021 – 2021/0106(COD). Available at: https://www.europarl.europa.eu/doceo/document/TA-9-2023-0236_EN.html (Accessed November 7, 2023).

[ref26] Fisher L. (2009). Target marketing of subprime loans: racialized consumer fraud and reverse redlining. Brooklyn Law J. 18, 121–155.

[ref27] Franklin M. Tomei P. Gorman R. (2023). Vague concepts in the EU AI act will not protect citizens from AI manipulation. OECD.AI policy observatory. Available at: https://oecd.ai/en/wonk/eu-ai-act-manipulation-definitions (Accessed November 7, 2023).

[ref28] Goldenberg A. Gross J. J. (2020). Digital emotion contagion. Trends Cogn. Sci. 24, 316–328. doi: 10.1016/j.tics.2020.01.009 32160568

[ref29] González-Bailón S. Lazer D. Barberá P. Zhang M. Alcott H. Brown T.. (2023). Asymmetric ideological segregation in exposure to political news on Facebook. Science 381, 392–398. doi: 10.1126/science.ade7138, PMID: 37499003

[ref30] Gregory S. (2023). Testimony of Sam Gregory, executive director, WITNESS before the United States house committee on oversight and accountability, subcommittee on cybersecurity, information technology, and government innovation ‘advances in Deepfake technology’. Available at: https://oversight.house.gov/wp-content/uploads/2023/11/Sam-Gregory-House-Oversight-Committee-Advances-in-Deepfake-Technology-November-2023.pdf (Accessed November 13, 2023).

[ref31] Guess A. M. Malhotra N. Pan J. Barberá P. Allcott H. Brown T.. (2023a). How do social media feed algorithms affect attitudes and behavior in an election campaign? Science 381, 398–404. doi: 10.1126/science.abp9364, PMID: 37498999

[ref32] Guess A. M. Malhotra N. Pan J. Barberá P. Allcott H. Brown T.. (2023b). Reshares on social media amplify political news but do not detectably affect beliefs or opinions. Science 381, 404–408. doi: 10.1126/science.add8424 37499012

[ref33] Hagey K. Horwitz J. (2021). Facebook tried to make its platform a healthier place. It got angrier instead. Wall street journal, September 15 2021. Available at: https://www.wsj.com/articles/facebook-algorithm-change-zuckerberg-11631654215?mod=article_inline (Accessed November 7, 2023).

[ref34] Hao K. (2021). How Facebook got addicted to spreading misinformation. MIT technology review. Available at: https://www.technologyreview.com/2021/03/11/1020600/facebook-responsible-ai-misinformation/ (Accessed November 7, 2023).

[ref35] Hochschild A. R. (1975). “The sociology of feeling and emotion: selected possibilities” in Another voice. eds. Millman M. Kanter R., vol. 45 (New York: Anchor), 280–307.

[ref36] Hoes E. Aitken B. Zhang J. Gackowski T. Wojcieszak M. (2023). Prominent misinformation interventions reduce misperceptions but increase skepticism. PsyArXiv Preprints [preprint]. doi: 10.31234/osf.io/zmpdu PMC11343704 38858544

[ref37] ICO. (2022). Biometrics: foresight. Information Commissioner’s office. Available at: https://ico.org.uk/media/4021971/biometrics-foresight-report.pdf (Accessed May 12, 2024).

[ref38] Jensen T. Vistisen P. (2017). “Ethical design fiction: between storytelling and world building”, In: ETHICOMP 2017 Conference Proceedings: Values in Emerging Science and Technology, 1, 15.

[ref9004] Kim Y. M. Hsu J. Neiman D. Kou C. Bankston L. Kim S. Y.. (2018). The stealth media? Groups and targets behind divisive issue campaigns on Facebook. Political Communication 35, 515–541. doi: 10.1080/10584609.2018.1476425

[ref39] Köbis N. Doležalová B. Soraperra I. (2021). Fooled twice – people cannot detect deepfakes but think they can. SSRN Electron. J. doi: 10.2139/ssrn.3832978 PMC8602050 34820608

[ref40] Laffer A. (2022). “Using an online narrative approach to explore diverse participants' understanding of emerging technology: citizen’s perspectives on living with emotional AI” in SAGE research methods: Doing online research (SAGE).

[ref41] Laffer A. (2023). Attitudes towards emotional artificial intelligence use: transcripts of citizen workshops collected using an innovative narrative approach, 2021. UK data service. [DATASET]. doi: 10.5255/UKDA-SN-855688

[ref42] Langguth J. Pogorelov K. Brenner S. Filkukovam P. Schroeder D. (2021). Don't trust your eyes: image manipulation in the age of DeepFakes. Front. Commun. 6:632317. doi: 10.3389/fcomm.2021.632317

[ref43] Layder D. (1998). Sociological practice: Linking theory and social research. London: Sage.

[ref44] Liv N. Greenbaum D. (2020). Deep fakes and memory malleability: false memories in the service of fake news. AJOB Neurosci. 11, 96–104. doi: 10.1080/21507740.2020.1740351, PMID: 32228386

[ref45] Luminate (2023). Bots versus ballots: Europeans fear AI threat to elections and lack of control over personal data. Available at: https://www.luminategroup.com/posts/news/bots-versus-ballots-europeans-fear-ai-threat-to-elections-and-lack-of-control-over-personal-data?utm_source=EURACTIV&utm_campaign=1110bd5411-EMAIL_CAMPAIGN_2023_09_15_09_23&utm_medium=email&utm_term=0_-1110bd5411-%5BLIST_EMAIL_ID%5D (Accessed November 7, 2023).

[ref46] Madden M. Gilman M. Levy K. Marwick A. (2017). Privacy, poverty, and big data: a matrix of vulnerabilities for poor Americans. Washington university law review 95: 053. Available at: https://openscholarship.wustl.edu/law_lawreview/vol95/iss1/6. (Accessed May 1, 2024)

[ref47] Mancini C. Rogers Y. Bandara A. K. Coe T. Jedrzejczyk L. Joinson A. N.. (2010). “ContraVision: exploring users’ reactions to futuristic technology”, In: Proceedings of the SIGCHI Conference on Human Factors in Computing Systems. 2010, 153–162.

[ref48] Mantello P. Ho M.-T. Nguyen M.-H. Vuong Q.-H. (2021). Bosses without a heart: socio-demographic and cross-cultural determinants of attitude toward emotional AI in the workplace. AI & Soc. 38, 97–119. doi: 10.1007/s00146-021-01290-1, PMID: 34776651 PMC8571983

[ref49] Martel C. Pennycook G. Rand D. G. (2020). Reliance on emotion promotes belief in fake news. Cogn. Res. Principles Implicat. 5, 1–20. doi: 10.31234/osf.io/a2ydw PMC7539247 33026546

[ref50] McNamee R. (2019). Zucked: Waking up to the Facebook catastrophe. Glasgow: Harper Collins.

[ref51] McStay A. (2014). Privacy and philosophy; new media and affective protocol. New York: Peter Lang.

[ref52] McStay A. (2016). Empathic media and advertising: industry, policy, legal and citizen perspectives (the case for intimacy). Big Data Soc. 3:205395171666686. doi: 10.1177/2053951716666868

[ref53] McStay A. (2018). Emotional AI: The rise of empathic media. London: Sage.

[ref54] McStay A. (2020a). Emotional AI, soft biometrics and the surveillance of emotional life: an unusual consensus on privacy. Big Data Soc. 7:205395172090438. doi: 10.1177/2053951720904386

[ref55] McStay A. (2020b). Emotional AI and edtech: serving the public good? Learn. Media Technol. 45, 270–283. doi: 10.1080/17439884.2020.1686016

[ref57] McStay A. (2023). Automating empathy: Decoding technologies that gauge intimate life. New York: Oxford University Press.

[ref58] McStay A. Bakir V. Urquhart L. Miranda D. (2023). Emotional AI survey, UK: aggregate data, 2022. UK data service. [DATASET]. doi: 10.5255/UKDA-SN-856708 PMC11190365 38912311

[ref59] McStay A. Rosner G. (2021). Emotional artificial intelligence in children’s toys and devices: ethics, governance and practical remedies. Big Data Soc. 8:205395172199487. doi: 10.1177/2053951721994877

[ref60] McStay A. Urquhart L. (2022a). ICO technology and innovation foresight call for views: biometric technologies. Emotional AI lab. Available at: https://drive.google.com/file/d/1J5v8thEECtodGvShySWTwVALd-DPIdBn/view (Accessed May 12, 2023).

[ref61] McStay A. Urquhart L. (2022b). In cars (are we really safest of all?): interior sensing and emotional opacity. Int. Rev. Law Comput. Technol. 36, 470–493. doi: 10.1080/13600869.2021.2009181

[ref62] Miles M. B. Huberman A. M. Saldana J. (2014). Qualitative data analysis. London: Sage.

[ref63] Nightingale S. J. Farid H. (2022). AI-synthesized faces are indistinguishable from real faces and more trustworthy. Proc. Natl. Acad. Sci. U.S.A. 119:e2120481119. doi: 10.1073/pnas.2120481119, PMID: 35165187 PMC8872790

[ref64] Nyhan B. Settle J. Thorson E. Wojcieszak M. Barberá P. Chen A. Y.. (2023). Like-minded sources on Facebook are prevalent but not polarizing. Nature 620, 137–144. doi: 10.1038/s41586-023-06297-w, PMID: 37500978 PMC10396953

[ref65] Ofcom (2023). User attitudes towards on-platform interventions: qualitative findings. YouGov qualitative. Available at: https://www.ofcom.org.uk/__data/assets/pdf_file/0020/270371/ofcom-interventions-qual-report.pdf (Accessed November 7, 2023).

[ref66] Ong J. C. Cabañes J. V. A. (2018). Architects of networked disinformation: behind the scenes of troll accounts and fake news production in the Philippines. doi: 10.7275/2cq4-5396

[ref67] Oremus W. Alcantara C. Merrill J. B. Galocha A. (2021). Facebook under fire: how Facebook shapes your feed. Washington post, October 26 2021. Available at: https://www.washingtonpost.com/technology/interactive/2021/how-facebook-algorithm-works/ (Accessed November 7, 2023).

[ref68] Papadogiannakis E. Papadopoulos P. Markatos E. P. Kourtellis N. (2022). Who funds misinformation? A systematic analysis of the ad-related profit routines of fake news sites. ArkXiv [preprint]. doi: 10.48550/arXiv.2202.05079

[ref69] Paschen J. (2019). Investigating the emotional appeal of fake news using artificial intelligence and human contributions. J. Product Brand Manag. 29, 223–233. doi: 10.1108/JPBM-12-2018-2179

[ref70] Rhue L. (2018). Racial influence on automated perceptions of emotions. Available at: https://ssrn.com/abstract=3281765 (Accessed November 7, 2023).

[ref71] Rog D. J. Bickman L. (2009). The SAGE handbook of applied social research methods. Thousand Oaks, California: SAGE.

[ref72] Sankin A. (2020). Facebook allows advertisers to target people who appear interested in pseudoscience. The markup, April 23. Available at: https://laist.com/news/facebook-pseudoscience-advertisers (Accessed November 7, 2023).

[ref73] Santini R. M. Salles D. Tucci G. (2021). When machine behavior targets future voters: the use of social bots to test narratives for political campaigns in Brazil. Int. J. Commun. 15, 1220–1243.

[ref74] Schneier B. (2020). Bots are destroying political discourse as we know it. The Atlantic, January 7 2020. Available at: https://www.theatlantic.com/technology/archive/2020/01/future-politics-bots-drowning-out-humans/604489/ (Accessed November 7, 2023).

[ref75] Skinner B. F. (2005[1948]). Walden Two. Indianapolis: Hackett Publishing Company Inc.

[ref76] Stark L. (2018). Algorithmic psychometrics and the scalable subject. Soc. Stud. Sci. 48, 204–231. doi: 10.1177/0306312718772094, PMID: 29726810

[ref77] Stark L. Hoey J. (2020). The ethics of emotion in artificial intelligence systems. OSF preprints. doi: 10.31219/osf.io/9ad4u

[ref78] Stark L. Hutson J. (2021). Physiognomic artificial intelligence. Available at: https://papers.ssrn.com/sol3/papers.cfm?abstract_id=3927300 (Accessed November 7, 2023).

[ref9005] Stella M. Ferrara E. De Domenico M. (2018). Bots increase exposure to negative and inflammatory content in online social systems. Proceedings of the National Academy of Sciences, 115, 12435–12440. doi: 10.1073/pnas.1803470115 PMC6298098 30459270

[ref79] Strong C. Owen K. Mansfield J. (2023). Understanding experiences of minority beliefs on online communication platforms. Ofcom.

[ref80] Sunstein C. R. (2016). The ethics of influence: Government in the age of Behavioural science. New York: Cambridge University Press.

[ref81] Susser D. Roessler B. Nissenbaum H. (2019). Online manipulation: hidden influences in a digital world. Georgetown Law Technol. Rev. 1, 1–45. doi: 10.2139/ssrn.3306006

[ref82] The British Academy. (2022). Understanding digital poverty and inequality in the UK. Digital society. Available at: https://www.thebritishacademy.ac.uk/publications/understanding-digital-poverty-and-inequality-in-the-uk/ (Accessed November 7, 2023).

[ref83] UNCRC (2013). The United Nations convention on the rights of the child. Available at: https://www.unicef.org.uk/wp-content/uploads/2016/08/unicef-convention-rights-child-uncrc.pdf (Accessed November 7, 2023).

[ref84] United Nations (2022). Countering disinformation for the promotion and protection of human rights and fundamental freedoms. A/77/287. Available at: https://documents-dds-ny.un.org/doc/UNDOC/GEN/N22/459/24/PDF/N2245924.pdf?OpenElement (Accessed November 7, 2023).

[ref85] Urquhart L. Laffer A. Miranda D. (2022). “Working with affective computing: exploring UK public perceptions of AI enabled workplace surveillance”, In: Ethicomp 2022 Proceedings, 165–177.

[ref86] Vaccari C. Chadwick A. (2020). Deepfakes and disinformation: exploring the impact of synthetic political video on deception, uncertainty, and trust in news. Soc. Media Soc. 6:205630512090340. doi: 10.1177/2056305120903408

[ref87] Vaccari C. Chadwick A. Kaiser J. (2022). The campaign disinformation divide: believing and sharing news in the 2019 UK general election. Polit. Commun. 40, 4–23. doi: 10.1080/10584609.2022.2128948

[ref88] Vaidhyanathan S. (2018). Antisocial media: How Facebook disconnects us and undermines democracy. New York: Oxford University Press.

[ref9006] Van Bavel J. J. Rathje S. Harris E. Robertson C. Sternisko A. (2021). How social media shapes polarization. Science & Society 25, 913–916. doi: 10.1016/j.tics.2021.07.013 34429255

[ref89] Von Otterlo M. (2014). Automated experimentation in Walden 3.0: the next step in profiling, predicting, control and surveillance. Surveil. Soc. 12, 255–272. doi: 10.24908/ss.v12i2.4600

[ref90] Vosoughi S. Roy D. Aral S. (2018). The spread of true and false news online. Science 359, 1146–1151. doi: 10.1126/science.aap9559, PMID: 29590045

[ref91] Walter N. Cohen J. Holbert R. L. Morag Y. (2020). Fact-checking: a meta-analysis of what works and for whom. Polit. Commun. 37, 350–375. doi: 10.1080/10584609.2019.1668894

[ref92] Wong J. C. (2019). Revealed: Facebook enables ads to target users interested in 'vaccine controversies. The Guardian, February 15, 2019. Available at: https://www.theguardian.com/technology/2019/feb/15/facebook-anti-vaccination-advertising-targeting-controversy (Accessed November 7, 2023).

[ref93] Zuboff S. (2019). The age of surveillance capitalism: The fight for a human future at the new frontier of power. New York: PublicAffairs Books.

